# Crystal structure and Hirshfeld surface analysis of 3-(3,5-di­meth­oxy­phen­yl)-5-[6-(1*H*-pyrazol-1-yl)pyridin-2-yl]-1*H*-1,2,4-triazole

**DOI:** 10.1107/S2056989025010977

**Published:** 2026-01-01

**Authors:** Maksym Seredyuk, Sergiu Shova, Nataliia S. Kariaka, Yurii S. Moroz, Dmitriy M. Panov, Oksana Tananaiko, Kateryna Znovjyak

**Affiliations:** a Department of Chemistry, Taras Shevchenko National University of Kyiv, Volodymyrska Street 64, Kyiv, 01601, Ukraine; b Department of Inorganic Polymers "Petru Poni" Institute of Macromolecular Chemistry Romanian Academy of Science Aleea Grigore Ghica Voda 41-A Iasi 700487 Romania; c ChemBioCenter Kyiv National Taras Shevchenko University Kyiv 02094 61 Winston Churchill Street Ukraine; Universidad de Los Andes Mérida, Venezuela

**Keywords:** crystal structure, tridentate ligands, triazole, pyridine, pyrazole

## Abstract

The title bis­azole­pyridine compound, C_18_H_16_N_6_O_2_, crystallizes in the triclinic space group *P*1 (No. 2) with two independent mol­ecules in the asymmetric unit. The mol­ecular structure was analyzed using crystallographic techniques, confirming the expected configuration and bonding scheme. Hirshfeld surface analysis revealed key non-covalent inter­actions such as C—H⋯N/C/O and π–π stacking, which consolidate the crystal structure. The study provides valuable insights into the structural features and inter­mol­ecular inter­actions of this polydentate compound, which may have potential applications as a transition-metal ligand.

## Abstract 2

The title asymmetric biazolpyridine crystallizes in the triclinic space group *P*1 with two independent mol­ecules in the asymmetric unit. Structural and Hirshfeld surface analysis revealed key non-covalent inter­actions such as C—H⋯N/C/O and π–π stacking, which consolidate the crystal structure.

## 1. Chemical context

Bisazole­pyridines, particularly tridentate ligands, are a highly versatile class of compounds with significant potential in functional materials, finding applications in biochemistry (Fares *et al.*, 2020 ▸), catalysis (Wei *et al.*, 2015 ▸), and mol­ecular magnetism (Halcrow, 2024 ▸). The strategic incorporation of substituents on their aromatic rings enables precise tuning of electronic, optical, and chemical properties, enhancing their utility while maintaining synthetic accessibility (Lu *et al.*, 2016 ▸). In mol­ecules featuring asymmetric architectures, such as those with an NH-bearing azole moiety, the formation of supra­molecular hydrogen-bonded networks of varying dimensionality is observed. The nature of peripheral substituents significantly influences inter­molecular inter­actions, thereby governing crystal packing, network connectivity, and the topology of these supra­molecular assemblies. Given the established importance of bis­azole­pyridines in coordination chemistry and our ongoing exploration of 3*d*-metal complexes with polydentate ligands (Piñeiro-López *et al.*, 2018 ▸; Seredyuk *et al.*, 2022 ▸, 2025 ▸), we herein report the crystal structure of an asymmetric tridentate ligand, 3-(3,5-di­meth­oxy­phen­yl)-5-[6-(1*H*-pyrazol-1-yl)pyridin-2-yl]-1*H*-1,2,4-triazole.
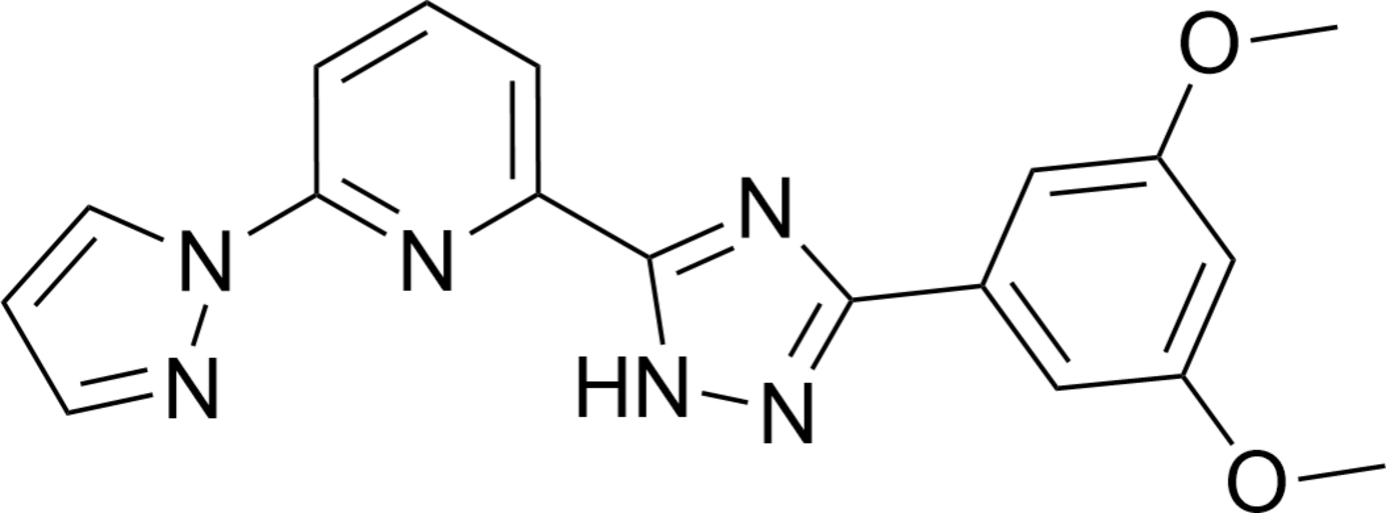


## 2. Structural commentary

**Figure 1:**
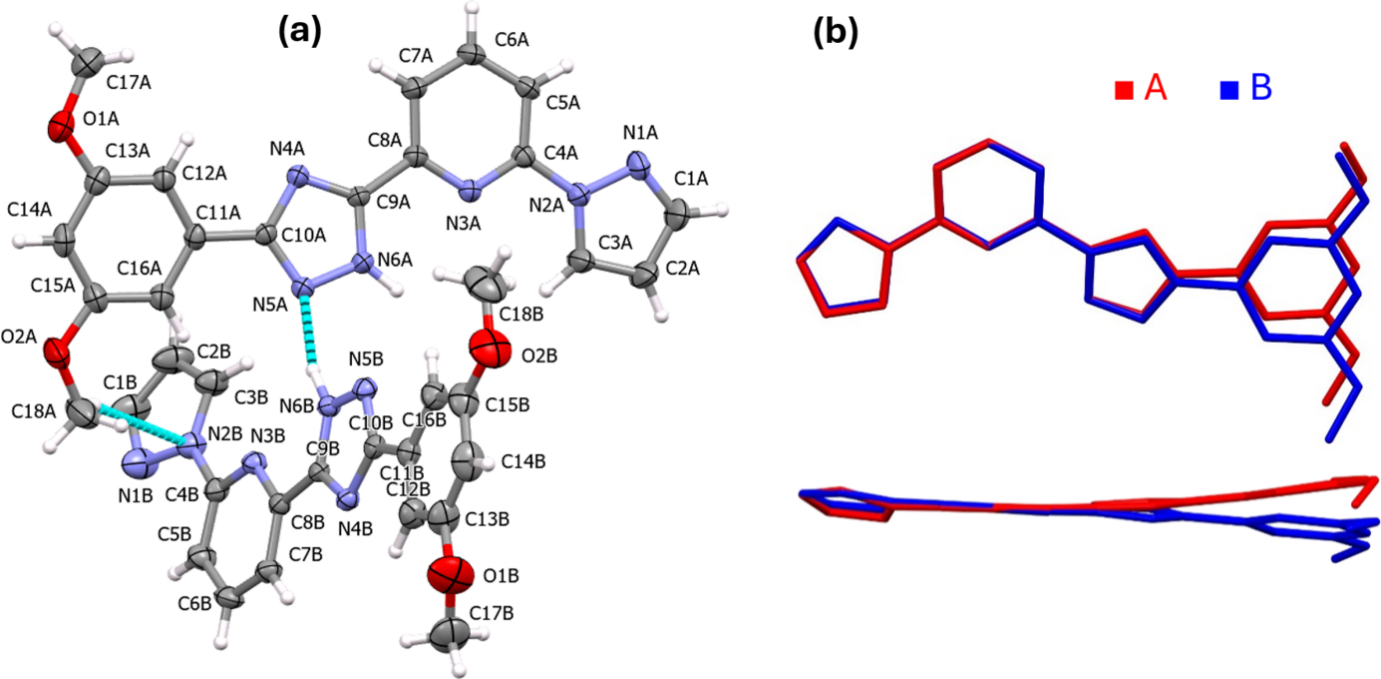
(*a*) The mol­ecular structure of mol­ecules *A* and *B* in the title compound with displacement ellipsoids drawn at the 40% probability level. The strong N—H⋯N and weak C—H⋯N hydrogen bonds are shown with the nearest neighbors. (*b*) Minimized overlay of the mol­ecules in two projections.

The title compound crystallizes in the triclinic system with space group *P*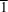 (No. 2), with two mol­ecules in the asymmetric unit (*Z* = 4). The mol­ecules, labeled *A* and *B*, have slightly different conformations and are labeled correlatively (Fig. 1 ▸*a*). The pyridine-triazole (py-trz) fragments in each mol­ecule are almost coplanar (r.m.s. deviation for *A*/*B* = 0.045/0.032 Å), except for the pyrazole (pz) and 3,5-di­meth­oxy­phenyl (ph) moieties in both cases. The dihedral angles between the py-trz and pz fragments are of 11.0 (3) and 17.8 (3)° for mol­ecules *A* and *B*, respectively. The ph fragment is rotated by 10.5 (3)° in mol­ecule *A*, whereas in mol­ecule *B* it is bent at an angle of 7.5 (3)° with respect to the py-trz plane. In both mol­ecules, the pz-py fragments exhibit an *anti* conformation, resulting in maximal spatial separation between the nitro­gen atoms N1*A*/*B* and N3*A*/*B* (Bessel *et al.*, 1992 ▸). Fig. 1 ▸*b* shows an overlay of mol­ecules *A* and *B* in two projections, visualizing the differences in their conformations.

## 3. Supra­molecular features

**Figure 2:**
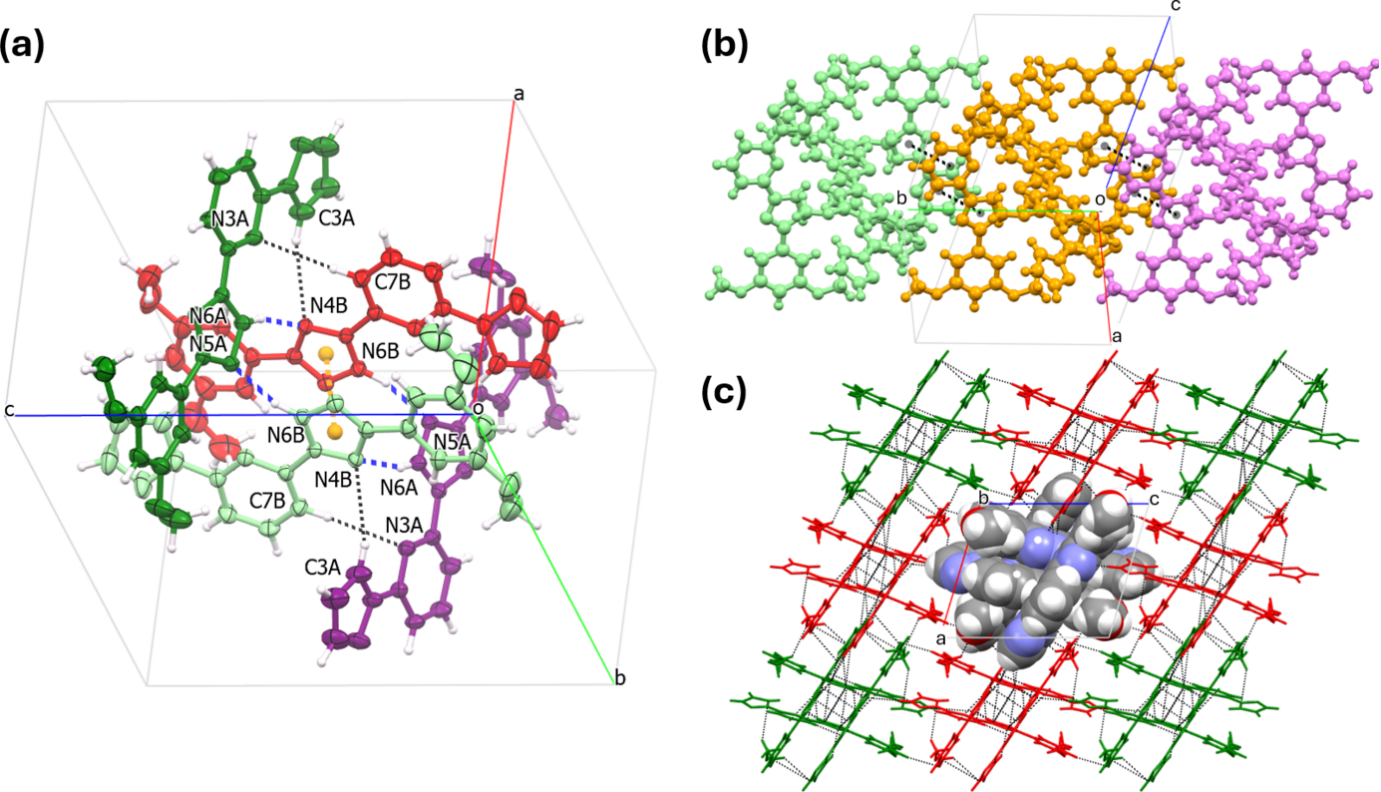
(*a*) Two pairs of mol­ecules *A* and *B* forming a circular fragment through strong N—H⋯N hydrogen bonds (blue dashed lines), weak C—H⋯N hydrogen bonds (black dashed lines) and π–π stacking of triazole rings (contact between centroids (orange globes) shown as dashed orange line). (*b*) A supra­molecular chain composed of tetra­molecular units. Only π–π stacking is shown for clarity. (*c*) A projection of the supra­molecular chains along the *b* axis. The inter­molecular contacts are shown as black dashed lines. For clarity, the central chain is shown with a space-filling model, while the surrounding chains are shown in capped-stick mode and colored differently.

In the crystal, two pairs of mol­ecules, *A* and *B*, form a cyclic supra­molecular tetra­molecular unit. They are joined through strong N—H⋯N′ hydrogen bonds, weak C—H⋯N′ bonds, and parallel, displaced π–π stacking between the trz rings of the neighbor mol­ecule *B* [*Cg*⋯*Cg*’ distance is 3.469 (4) Å] (Fig. 2 ▸*a*). Neighboring tetra­molecular units are united into supra­molecular chains along the *b* axis (Fig. 2 ▸*b*) *via* π–π stacking inter­actions between the coplanar trz and py rings of neighboring mol­ecules *A* [*Cg*(trz)⋯*Cg*(py)’ distance is 3.883 (4) Å], and weak C—H⋯N/C hydrogen bonds. At the highest level of organization, the supra­molecular chains are linked into a three-dimensional network through C—H⋯O/N and C⋯C inter­molecular contacts. All relevant inter­molecular contacts are collected in Table 1 ▸.

**Figure 3:**
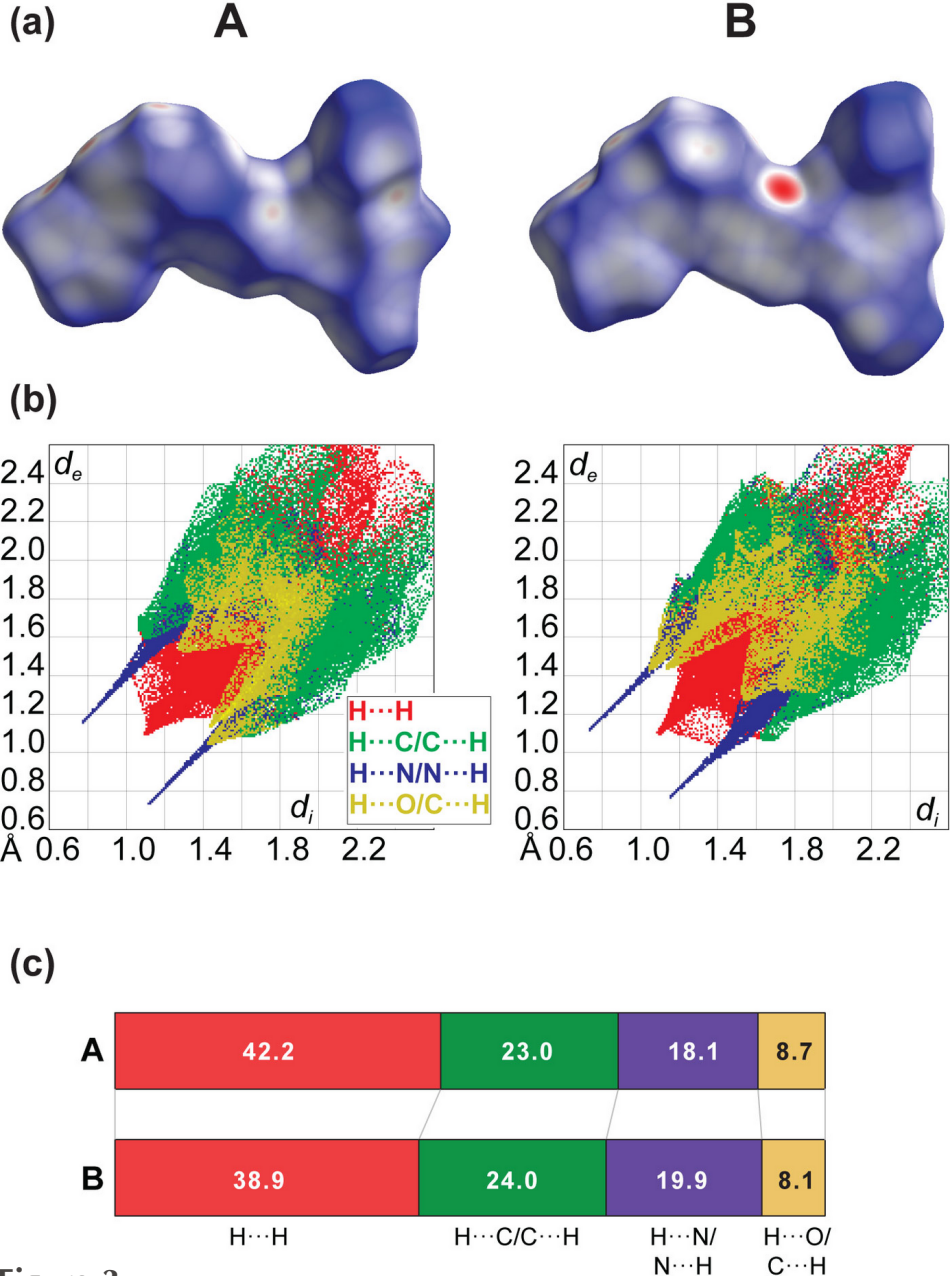
(*a*) Overall Hirshfeld surface for mol­ecules *A* and *B* and (*b*) their respective two-dimensional fingerprint plots decomposed into specific inter­actions. (*c*) Comparison of the contributions in mol­ecules *A* and *B*.

Hirshfeld surface analysis was conducted on each mol­ecule individually to gain a deeper understanding of inter­molecular inter­actions (Spackman *et al.*, 2021 ▸). The inter­actions are visualized as red (*d*_norm_ < vdW radii), white (*d*_norm_ = vdW radii), and blue (*d*_norm_ > vdW radii) spots on the *d*_norm_ surfaces for all compounds, alongside with fingerprint plots mapped with *d*_norm_ (where *d*_norm_* = d*_i_ + *d*_e_) (Fig. 3 ▸*a*). Two-dimensional fingerprint plots, with the relative contributions of individual contacts to the Hirshfeld surface mapped over *d*_norm_, are shown in Fig. 3 ▸*b*. At *ca*. 40%, the largest contribution to the overall crystal packing is from H⋯H inter­actions, which are in the middle region of the fingerprint plot. C⋯H/H⋯C contacts contribute *ca*. 24%, and N⋯H/H⋯N *ca*. 19%, resulting in pairs of characteristic sharp spikes. The O⋯H/H⋯O contacts, represented by a pair of wings in the inner part of the fingerprint plot, make *ca*. 8% contribution to the surface. Fig. 3 ▸*c* shows a comparison of the percentage contribution of contacts to the Hirshfeld surface for the two molecules.

## 4. Database survey

A search of the Cambridge Structural Database (CSD, Version 5.42, last update April 2025; Groom *et al.*, 2016 ▸) reveals 360 free bis­azole­pyridine ligands. For pyrazole- and 1,2,3-triazole-based mol­ecules, the *anti*-conformation of the azole-pyridine fragment is observed, reducing steric hindrance due to hydrogen crowding [KALXIG (Roberts *et al.*, 2012 ▸); IJOJAU (Byrne *et al.*, 2016 ▸)]. Various supra­molecular networks formed by strong hydrogen bonding have been identified for azoles with an unsubstituted NH group, including clusters (ABUFIP and ABUGIQ; Rajnak *et al.*, 2017 ▸) and one-dimensional supra­molecular chains [ABUGEM (Rájnak *et al.*, 2017 ▸); PUWZAJ (Craig *et al.*, 2010 ▸); QETVEQ (Pleier *et al.*, 2001 ▸); WEJGAU (Demir *et al.*, 2006 ▸), and XOMJIW (Le-Hoang *et al.*, 2024 ▸)] found in asymmetrically substituted bis­benzimidazole­pyridines and symmetric bis­pyrazolyl­pyridines. Additionally, a three-dimensional network, supported by strong hydrogen bonding, has been described for a compact mol­ecule bis­dihydro­imidazole­pyridine (BIHLAH; Geden *et al.*, 2013 ▸).

## 5. Synthesis and crystallization

The ligand was synthesized by a modified procedure reported earlier (Seredyuk *et al.*, 2022 ▸). All chemicals were purchased from commercial suppliers and used without further purification (Merck, Enamine Ltd).

A Schlenk flask with inert atmosphere was charged with 6-(1*H*-pyrazol-1-yl)pyridin-2-ylboronic acid (1.00 g, 5.3 mmol), 5-bromo-3-(3,5-di­meth­oxy­phen­yl)-1-(tetra­hydro-2*H*-pyran-2-yl)-1*H*-1,2,4-triazole (1.37 g, 4.8 mmol), [Pd(PPh_3_)_4_] (0.61 g, 0.53 mmol) and Na_2_CO_3_ (1.65 g, 15.6 mmol). Degassed 1,4-dioxane (20 mL) and degassed water (10 mL) were added, and the reaction mixture was heated to 373 K under vigorous stirring for 16 h. After filtering through a Celite pad, to the obtained solution 5 ml of HCl_aq_ (37%) was added dropwise and the obtained solution was stirred for 10 min. Thereafter the pH of the solution was brought to neutral with an aqueous solution of NaOH (10%). The resulting suspension was evaporated to dryness and resuspended in water, the precipitate was collected by filtration, washed with water and recrystallized from chloro­form-acetone (1:1). After drying *in vacuo*, the final compound was isolated as an analytically pure white crystalline material. Yield: 1.24 g, 67%. Elemental analysis calculated for C_18_H_16_N_6_O_2_: C, 62.06; H, 4.63; N, 24.12. Found: C, 62.24; H, 4.54; N, 24.01. ^1^H NMR (300 MHz, 298 K, DMSO-*d*^6^): δ (ppm) 14.92 (1H, *s*, trzH), 9.01 (1H, *s*, pzH), 8.11–8.02 (3H, *m*, pyH), 7.80 (1H, *s*, pzH), 7.64 (3H, *m*, pzH, phH), 6.60 (1H, *s*, pzH), 6.38 (1H, *s*, phH), 3.85 (6H, *s*, CH_3_). ^13^C NMR (100 MHz, DMSO-*d*^6^): δ (ppm) 161.1, 154.9, 154.1, 149.2, 148.0, 141.4, 129.4, 126.3, 126.5, 118.6, 110.6, 107.6, 107.2, 99.7, 55.3.

## 6. Refinement

Crystal data, data collection and structure refinement details are summarized in Table 2 ▸. H atoms were refined as riding [C—H = 0.93–0.96 Å with *U*_iso_(H) = 1.2–1.5*U*_eq_(C)]. The hydrogen atoms H6*A* and H6*B* were refined freely.

## Supplementary Material

Crystal structure: contains datablock(s) I. DOI: 10.1107/S2056989025010977/dj2085sup1.cif

Structure factors: contains datablock(s) I. DOI: 10.1107/S2056989025010977/dj2085Isup2.hkl

Supporting information file. DOI: 10.1107/S2056989025010977/dj2085Isup3.cdx

Supporting information file. DOI: 10.1107/S2056989025010977/dj2085Isup4.cml

CCDC reference: 2513428

Additional supporting information:  crystallographic information; 3D view; checkCIF report

## Figures and Tables

**Table 1:** Hydrogen-bond and short contact geometry (Å, °)

Contact	*H⋯A*	*D⋯A*	*D—H⋯A*
C7*A*—H⋯C6*B*^i^	2.89	3.820 (3)	171
C7*A*—H⋯C7*B*^i^	2.87	3.761 (3)	160
N6*B*—H⋯N5*A*	1.88 (3)	2.841 (3)	162 (2)
C18*A*—H⋯N2*B*	2.82	3.591 (3)	137
C14*B*^ii^—H^ii^⋯O1*A*	2.64	3.559 (3)	168
C1*A*—H⋯N5*B*^iii^	2.47	3.314 (3)	150
C1*A*—H⋯N6*B*^iii^	2.77	3.402 (3)	126
C17*B*^iv^—H^iv^⋯O2*A*^iv^	2.66	3.371 (4)	131
C6*A*—H⋯N5*B*^v^	2.62	3.524 (3)	165
C17*A*^v^—H^v^⋯C1*A*	2.79	3.490 (3)	131
C7*A*⋯C9*A*^v^	–	3.537 (2)	–
C7*B*—H⋯N3*A*^vi^	2.67	3.602 (3)	177
C3*A*^vi^—H^vi^⋯N4*B*	2.76	3.624 (3)	155
N6*A*^vi^—H^vi^⋯N4*B*	2.04 (3)	2.922 (3)	164 (2)
N5*B*⋯C9*B*^vi^	–	3.281 (3)	–
N6*B*⋯C9*B*^vi^	–	3.350 (3)	–
C1*B*—H⋯N4*A*^vii^	2.69	3.599 (3)	165
C14*A*⋯C5*A*^viii^	–	3.437 (2)	–
C5*A*^ix^—H^ix^⋯N1*A*	2.52	3.407 (3)	159
C5*B*^*x*^—H^*x*^⋯N1*B*	2.62	3.434 (2)	147

Symmetry codes: (i) *x*, 1 + *y*, *z*; (ii) *x*, *y*, 1 + *z*; (iii) −1 + *x*, *y*, *z*; (iv) −*x*, 1 − *y*, 1 − *z*; (v) 1 − *x*, −*y*, 1 − *z*; (vi) 1 − *x*, 1 − *y*, 1 − *z*; (vii) 1 − *x*, 1 − *y*, 2 − *z*; (viii) −1 + *x*, *y*, *z*; (ix) 2 − *x*, −*y*, 1 − *z*; (*x*) 1 − *x*, 2 − *y*, 2 − *z*.

**Table 2:** Experimental details

Crystal data	
Chemical formula	C_18_H_16_N_6_O_2_
*M*_r_	348.37
Crystal system, space group	Triclinic, *P* 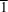
Temperature (K)	293
*a*, *b*, *c* (Å)	11.4607 (7), 12.0769 (7), 14.6724 (6)
α, β, γ (°)	114.104 (5), 98.689 (4), 104.469 (5)
*V* (Å^3^)	1719.76 (18)
*Z*	4
Radiation type	Mo *K*α
μ (mm^−1^)	0.09
Crystal size (mm)	0.2 × 0.1 × 0.05

Data collection	
Diffractometer	Xcalibur, Eos
Absorption correction	Multi-scan (*CrysAlis PRO*; Rigaku OD, 2024 ▸)
*T*_min_, *T*_max_	0.898, 1.000
No. of measured, independent and observed [*I* > 2σ(*I*)] reflections	16576, 6091, 4491
*R*_int_	0.036
(sin θ/λ)_max_ (Å^−1^)	0.595

Refinement	
*R*[*F*^2^ > 2σ(*F*^2^)], *wR*(*F*^2^), *S*	0.049, 0.115, 1.02
No. of reflections	6091
No. of parameters	486
H-atom treatment	H atoms treated by a mixture of independent and constrained refinement
Δρ_max_, Δρ_min_ (e Å^−3^)	0.20, −0.19

Computer programs: *CrysAlis PRO* (Rigaku OD, 2024 ▸), *SHELXT2018/2* (Sheldrick, 2015*a* ▸), *SHELXL2018/3* (Sheldrick, 2015*b* ▸) and *OLEX2* (Dolomanov *et al.*, 2009 ▸).

## supplementary crystallographic information

### 3-(3,5-Dimethoxyphenyl)-5-[6-(1H-pyrazol-1-yl)pyridin-2-yl]-1H-1,2,4-triazole Crystal data

**Table:**

C_18_H_16_N_6_O_2_	*Z* = 4
*M**_r_* = 348.37	*F*(000) = 728
Triclinic, *P*1	*D*_x_ = 1.345 Mg m^−^^3^
*a* = 11.4607 (7) Å	Mo *K*α radiation, λ = 0.71073 Å
*b* = 12.0769 (7) Å	Cell parameters from 4514 reflections
*c* = 14.6724 (6) Å	θ = 1.9–25.9°
α = 114.104 (5)°	µ = 0.09 mm^−^^1^
β = 98.689 (4)°	*T* = 293 K
γ = 104.469 (5)°	Prism, clear colourless
*V* = 1719.76 (18) Å^3^	0.2 × 0.1 × 0.05 mm

### 3-(3,5-Dimethoxyphenyl)-5-[6-(1H-pyrazol-1-yl)pyridin-2-yl]-1H-1,2,4-triazole Data collection

**Table:**

Xcalibur, Eos diffractometer	6091 independent reflections
Radiation source: fine-focus sealed X-ray tube, Enhance (Mo) X-ray Source	4491 reflections with *I* > 2σ(*I*)
Graphite monochromator	*R*_int_ = 0.036
Detector resolution: 16.1593 pixels mm^-1^	θ_max_ = 25.0°, θ_min_ = 1.9°
ω scans	*h* = −12→13
Absorption correction: multi-scan (CrysAlisPro; Rigaku OD, 2024)	*k* = −14→14
*T*_min_ = 0.898, *T*_max_ = 1.000	*l* = −17→16
16576 measured reflections	

### 3-(3,5-Dimethoxyphenyl)-5-[6-(1H-pyrazol-1-yl)pyridin-2-yl]-1H-1,2,4-triazole Refinement

**Table:**

Refinement on *F*^2^	Hydrogen site location: mixed
Least-squares matrix: full	H atoms treated by a mixture of independent and constrained refinement
*R*[*F*^2^ > 2σ(*F*^2^)] = 0.049	*w* = 1/[σ^2^(*F*_o_^2^) + (0.0486*P*)^2^ + 0.1128*P*] where *P* = (*F*_o_^2^ + 2*F*_c_^2^)/3
*wR*(*F*^2^) = 0.115	(Δ/σ)_max_ < 0.001
*S* = 1.02	Δρ_max_ = 0.20 e Å^−^^3^
6091 reflections	Δρ_min_ = −0.19 e Å^−^^3^
486 parameters	Extinction correction: SHELXL-2018/3 (Sheldrick, 2015b), Fc^*^=kFc[1+0.001xFc^2^λ^3^/sin(2θ)]^-1/4^
0 restraints	Extinction coefficient: 0.0110 (11)
Primary atom site location: dual	

### 3-(3,5-Dimethoxyphenyl)-5-[6-(1H-pyrazol-1-yl)pyridin-2-yl]-1H-1,2,4-triazole Special details

**Table:**

Geometry. All esds (except the esd in the dihedral angle between two l.s. planes) are estimated using the full covariance matrix. The cell esds are taken into account individually in the estimation of esds in distances, angles and torsion angles; correlations between esds in cell parameters are only used when they are defined by crystal symmetry. An approximate (isotropic) treatment of cell esds is used for estimating esds involving l.s. planes.

### 3-(3,5-Dimethoxyphenyl)-5-[6-(1H-pyrazol-1-yl)pyridin-2-yl]-1H-1,2,4-triazole Fractional atomic coordinates and isotropic or equivalent isotropic displacement parameters (Å^2^)

**Table:**

	*x*	*y*	*z*	*U*_iso_*/*U*_eq_	
O1B	0.1147 (2)	0.4019 (2)	0.05223 (12)	0.0876 (6)	
O2B	0.16741 (19)	0.02591 (16)	0.03701 (12)	0.0839 (6)	
N1B	0.4802 (2)	0.81432 (17)	0.99412 (12)	0.0626 (6)	
N2B	0.48491 (16)	0.73258 (15)	0.89884 (11)	0.0410 (4)	
N3B	0.42372 (14)	0.65010 (13)	0.72014 (10)	0.0317 (4)	
N4B	0.31820 (14)	0.53416 (13)	0.44042 (10)	0.0299 (4)	
N5B	0.33490 (15)	0.34712 (14)	0.42911 (11)	0.0327 (4)	
N6B	0.37109 (15)	0.43949 (14)	0.53020 (11)	0.0296 (4)	
C1B	0.5221 (3)	0.7683 (2)	1.05491 (17)	0.0702 (8)	
H1B	0.529704	0.804356	1.125876	0.084*	
C2B	0.5536 (2)	0.6608 (2)	1.00199 (17)	0.0633 (7)	
H2B	0.585488	0.613603	1.029255	0.076*	
C3B	0.5281 (2)	0.6395 (2)	0.90235 (16)	0.0481 (6)	
C4B	0.44583 (18)	0.75091 (17)	0.81179 (13)	0.0339 (5)	
C5B	0.4332 (2)	0.86710 (18)	0.82482 (14)	0.0434 (5)	
H5B	0.449034	0.934871	0.890762	0.052*	
C6B	0.3963 (2)	0.87811 (18)	0.73608 (14)	0.0441 (5)	
H6BA	0.387234	0.954708	0.741347	0.053*	
C7B	0.37285 (19)	0.77551 (17)	0.63930 (14)	0.0371 (5)	
H7B	0.347924	0.781626	0.578705	0.045*	
C8B	0.38729 (17)	0.66440 (17)	0.63500 (13)	0.0288 (4)	
C9B	0.36058 (17)	0.54910 (16)	0.53603 (13)	0.0264 (4)	
C10B	0.30287 (17)	0.40805 (17)	0.37766 (13)	0.0295 (4)	
C11B	0.25053 (18)	0.34309 (18)	0.26363 (13)	0.0355 (5)	
C12B	0.2098 (2)	0.4096 (2)	0.21513 (14)	0.0445 (5)	
H12B	0.217610	0.495589	0.253788	0.053*	
C13B	0.1569 (2)	0.3453 (2)	0.10762 (16)	0.0554 (6)	
C14B	0.1431 (2)	0.2171 (2)	0.05117 (17)	0.0630 (7)	
H14B	0.106114	0.174400	−0.020526	0.076*	
C15B	0.1836 (2)	0.1516 (2)	0.10019 (16)	0.0547 (6)	
C16B	0.2384 (2)	0.21444 (18)	0.20684 (14)	0.0431 (5)	
H16B	0.266878	0.171052	0.240084	0.052*	
C17B	0.1646 (3)	0.5366 (3)	0.0948 (2)	0.0842 (9)	
H17A	0.132522	0.562432	0.045319	0.126*	
H17B	0.254655	0.563892	0.111143	0.126*	
H17C	0.140921	0.575891	0.157215	0.126*	
C18B	0.2030 (3)	−0.0481 (2)	0.0822 (2)	0.0863 (9)	
H18A	0.182870	−0.135033	0.028885	0.130*	
H18B	0.158482	−0.048627	0.132528	0.130*	
H18C	0.291857	−0.010911	0.115975	0.130*	
H6B	0.395 (2)	0.4149 (19)	0.5855 (15)	0.061 (6)*	
O1A	0.03689 (14)	0.02853 (15)	0.77513 (11)	0.0559 (4)	
O2A	0.08246 (15)	0.43797 (15)	0.80954 (13)	0.0641 (5)	
N1A	0.99881 (17)	0.16289 (17)	0.50128 (13)	0.0488 (5)	
N2A	0.91260 (15)	0.21320 (15)	0.53939 (12)	0.0370 (4)	
N3A	0.73110 (14)	0.19317 (14)	0.59038 (11)	0.0324 (4)	
N4A	0.44916 (15)	0.15161 (15)	0.67608 (11)	0.0340 (4)	
N5A	0.44406 (15)	0.32577 (14)	0.65448 (11)	0.0337 (4)	
N6A	0.54281 (16)	0.29073 (15)	0.62779 (11)	0.0330 (4)	
C1A	1.0711 (2)	0.2539 (2)	0.48690 (19)	0.0608 (7)	
H1A	1.139375	0.246934	0.460693	0.073*	
C2A	1.0346 (2)	0.3612 (2)	0.51495 (19)	0.0612 (7)	
H2A	1.072018	0.436654	0.511611	0.073*	
C3A	0.9329 (2)	0.3327 (2)	0.54827 (17)	0.0511 (6)	
H3A	0.885887	0.385297	0.572625	0.061*	
C4A	0.81869 (18)	0.14176 (17)	0.56613 (13)	0.0312 (4)	
C5A	0.82266 (19)	0.02839 (18)	0.56628 (14)	0.0372 (5)	
H5A	0.885761	−0.004117	0.547796	0.045*	
C6A	0.7294 (2)	−0.03389 (19)	0.59488 (15)	0.0441 (5)	
H6AA	0.728504	−0.110383	0.596316	0.053*	
C7A	0.6366 (2)	0.01680 (18)	0.62167 (15)	0.0409 (5)	
H7A	0.573135	−0.024349	0.641693	0.049*	
C8A	0.64055 (18)	0.12961 (17)	0.61790 (13)	0.0321 (4)	
C9A	0.54420 (17)	0.18829 (17)	0.64107 (13)	0.0299 (4)	
C10A	0.39036 (18)	0.23937 (17)	0.68339 (13)	0.0303 (4)	
C11A	0.27718 (18)	0.23807 (18)	0.72001 (13)	0.0329 (5)	
C12A	0.21221 (19)	0.13060 (19)	0.72727 (13)	0.0370 (5)	
H12A	0.238919	0.059782	0.708326	0.044*	
C13A	0.10663 (19)	0.12898 (19)	0.76308 (14)	0.0396 (5)	
C14A	0.0666 (2)	0.2336 (2)	0.78920 (15)	0.0457 (5)	
H14A	−0.004613	0.232059	0.812416	0.055*	
C15A	0.1314 (2)	0.3404 (2)	0.78118 (15)	0.0428 (5)	
C16A	0.23827 (19)	0.34472 (19)	0.74742 (14)	0.0394 (5)	
H16A	0.283019	0.417301	0.743175	0.047*	
C17A	0.0922 (3)	−0.0640 (2)	0.77716 (19)	0.0647 (7)	
H17D	0.102934	−0.110880	0.710027	0.097*	
H17E	0.038350	−0.123174	0.793991	0.097*	
H17F	0.172604	−0.020493	0.828837	0.097*	
C18A	0.1532 (3)	0.5582 (2)	0.8185 (2)	0.0809 (9)	
H18D	0.164849	0.546260	0.752116	0.121*	
H18E	0.233705	0.592066	0.868743	0.121*	
H18F	0.108984	0.617809	0.840737	0.121*	
H6A	0.598 (2)	0.3392 (19)	0.6086 (14)	0.049 (6)*	
H3B	0.5344 (19)	0.5742 (19)	0.8403 (15)	0.056 (6)*	

### 3-(3,5-Dimethoxyphenyl)-5-[6-(1H-pyrazol-1-yl)pyridin-2-yl]-1H-1,2,4-triazole Atomic displacement parameters (Å^2^)

**Table:**

	*U*^11^	*U*^22^	*U*^33^	*U*^12^	*U*^13^	*U*^23^
O1B	0.1199 (18)	0.0981 (15)	0.0450 (10)	0.0487 (14)	−0.0009 (10)	0.0354 (10)
O2B	0.1142 (17)	0.0526 (11)	0.0466 (10)	0.0201 (11)	0.0101 (10)	−0.0028 (8)
N1B	0.1075 (18)	0.0449 (11)	0.0275 (10)	0.0269 (12)	0.0149 (10)	0.0107 (9)
N2B	0.0568 (12)	0.0340 (9)	0.0261 (9)	0.0133 (9)	0.0070 (8)	0.0114 (7)
N3B	0.0375 (10)	0.0280 (8)	0.0288 (8)	0.0112 (8)	0.0085 (7)	0.0130 (7)
N4B	0.0353 (10)	0.0305 (8)	0.0277 (8)	0.0123 (8)	0.0100 (7)	0.0163 (7)
N5B	0.0381 (10)	0.0281 (8)	0.0309 (8)	0.0099 (8)	0.0110 (7)	0.0135 (7)
N6B	0.0364 (10)	0.0266 (8)	0.0277 (8)	0.0115 (7)	0.0094 (7)	0.0140 (7)
C1B	0.112 (2)	0.0558 (15)	0.0302 (12)	0.0194 (16)	0.0058 (13)	0.0191 (11)
C2B	0.086 (2)	0.0533 (15)	0.0462 (13)	0.0188 (14)	0.0000 (13)	0.0282 (12)
C3B	0.0594 (16)	0.0453 (13)	0.0384 (12)	0.0191 (12)	0.0056 (11)	0.0207 (11)
C4B	0.0397 (12)	0.0313 (10)	0.0295 (10)	0.0112 (9)	0.0096 (9)	0.0138 (9)
C5B	0.0595 (15)	0.0328 (11)	0.0336 (11)	0.0179 (11)	0.0131 (10)	0.0106 (9)
C6B	0.0584 (15)	0.0308 (11)	0.0476 (12)	0.0225 (11)	0.0163 (11)	0.0181 (10)
C7B	0.0459 (13)	0.0359 (11)	0.0358 (10)	0.0170 (10)	0.0116 (9)	0.0207 (9)
C8B	0.0288 (11)	0.0304 (10)	0.0295 (10)	0.0103 (9)	0.0092 (8)	0.0159 (8)
C9B	0.0271 (11)	0.0264 (9)	0.0299 (10)	0.0093 (8)	0.0103 (8)	0.0162 (8)
C10B	0.0274 (11)	0.0313 (10)	0.0307 (10)	0.0085 (9)	0.0104 (8)	0.0155 (8)
C11B	0.0313 (12)	0.0401 (11)	0.0292 (10)	0.0080 (10)	0.0079 (8)	0.0136 (9)
C12B	0.0496 (14)	0.0500 (13)	0.0311 (11)	0.0177 (11)	0.0083 (10)	0.0171 (10)
C13B	0.0565 (16)	0.0734 (17)	0.0371 (12)	0.0248 (14)	0.0075 (11)	0.0270 (12)
C14B	0.0656 (18)	0.0712 (17)	0.0305 (12)	0.0165 (15)	0.0050 (11)	0.0108 (12)
C15B	0.0560 (16)	0.0461 (13)	0.0388 (13)	0.0086 (12)	0.0112 (11)	0.0046 (11)
C16B	0.0475 (14)	0.0387 (11)	0.0363 (11)	0.0117 (11)	0.0125 (10)	0.0127 (10)
C17B	0.095 (2)	0.114 (3)	0.0806 (19)	0.052 (2)	0.0272 (17)	0.0696 (19)
C18B	0.095 (2)	0.0458 (15)	0.0837 (19)	0.0199 (16)	0.0171 (17)	0.0042 (14)
O1A	0.0510 (10)	0.0606 (10)	0.0693 (10)	0.0177 (9)	0.0311 (8)	0.0387 (8)
O2A	0.0587 (11)	0.0578 (10)	0.0918 (12)	0.0338 (9)	0.0443 (10)	0.0339 (9)
N1A	0.0402 (11)	0.0499 (11)	0.0694 (12)	0.0243 (10)	0.0304 (9)	0.0299 (10)
N2A	0.0329 (10)	0.0375 (9)	0.0518 (10)	0.0178 (8)	0.0192 (8)	0.0255 (8)
N3A	0.0322 (10)	0.0343 (9)	0.0408 (9)	0.0154 (8)	0.0160 (7)	0.0226 (7)
N4A	0.0359 (10)	0.0400 (9)	0.0397 (9)	0.0186 (8)	0.0186 (8)	0.0251 (8)
N5A	0.0378 (10)	0.0381 (9)	0.0395 (9)	0.0208 (8)	0.0190 (8)	0.0242 (8)
N6A	0.0361 (10)	0.0356 (9)	0.0417 (9)	0.0179 (8)	0.0199 (8)	0.0252 (8)
C1A	0.0424 (15)	0.0655 (16)	0.0958 (18)	0.0223 (13)	0.0405 (14)	0.0481 (15)
C2A	0.0481 (16)	0.0561 (15)	0.1067 (19)	0.0204 (13)	0.0396 (14)	0.0556 (15)
C3A	0.0449 (15)	0.0448 (13)	0.0844 (16)	0.0237 (12)	0.0311 (12)	0.0403 (12)
C4A	0.0306 (12)	0.0332 (10)	0.0326 (10)	0.0134 (9)	0.0103 (8)	0.0160 (8)
C5A	0.0384 (13)	0.0354 (11)	0.0472 (11)	0.0214 (10)	0.0162 (10)	0.0218 (9)
C6A	0.0504 (15)	0.0371 (11)	0.0619 (13)	0.0241 (11)	0.0231 (11)	0.0310 (10)
C7A	0.0430 (13)	0.0382 (11)	0.0580 (13)	0.0186 (10)	0.0236 (10)	0.0317 (10)
C8A	0.0338 (12)	0.0331 (10)	0.0357 (10)	0.0146 (9)	0.0118 (9)	0.0195 (9)
C9A	0.0343 (12)	0.0318 (10)	0.0328 (10)	0.0153 (9)	0.0140 (9)	0.0198 (8)
C10A	0.0331 (11)	0.0355 (10)	0.0299 (10)	0.0151 (9)	0.0116 (8)	0.0195 (8)
C11A	0.0304 (12)	0.0426 (11)	0.0298 (10)	0.0137 (10)	0.0107 (8)	0.0193 (9)
C12A	0.0380 (13)	0.0443 (12)	0.0377 (11)	0.0193 (10)	0.0169 (9)	0.0223 (9)
C13A	0.0385 (13)	0.0451 (12)	0.0353 (11)	0.0101 (11)	0.0126 (9)	0.0206 (10)
C14A	0.0357 (13)	0.0598 (14)	0.0455 (12)	0.0194 (12)	0.0210 (10)	0.0232 (11)
C15A	0.0408 (13)	0.0472 (12)	0.0429 (12)	0.0214 (11)	0.0182 (10)	0.0178 (10)
C16A	0.0399 (13)	0.0433 (12)	0.0410 (11)	0.0171 (10)	0.0167 (10)	0.0219 (9)
C17A	0.0735 (19)	0.0612 (16)	0.0781 (17)	0.0233 (15)	0.0376 (14)	0.0441 (14)
C18A	0.078 (2)	0.0544 (16)	0.122 (2)	0.0366 (16)	0.0506 (18)	0.0363 (16)

### 3-(3,5-Dimethoxyphenyl)-5-[6-(1H-pyrazol-1-yl)pyridin-2-yl]-1H-1,2,4-triazole Geometric parameters (Å, º)

**Table:**

O1B—C13B	1.365 (3)	O1A—C13A	1.369 (2)
O1B—C17B	1.400 (3)	O1A—C17A	1.424 (3)
O2B—C15B	1.362 (3)	O2A—C15A	1.370 (2)
O2B—C18B	1.410 (3)	O2A—C18A	1.417 (3)
N1B—N2B	1.362 (2)	N1A—N2A	1.359 (2)
N1B—C1B	1.321 (3)	N1A—C1A	1.314 (3)
N2B—C3B	1.352 (2)	N2A—C3A	1.350 (2)
N2B—C4B	1.410 (2)	N2A—C4A	1.413 (2)
N3B—C4B	1.327 (2)	N3A—C4A	1.326 (2)
N3B—C8B	1.349 (2)	N3A—C8A	1.346 (2)
N4B—C9B	1.334 (2)	N4A—C9A	1.327 (2)
N4B—C10B	1.368 (2)	N4A—C10A	1.369 (2)
N5B—N6B	1.3593 (19)	N5A—N6A	1.361 (2)
N5B—C10B	1.323 (2)	N5A—C10A	1.329 (2)
N6B—C9B	1.328 (2)	N6A—C9A	1.332 (2)
N6B—H6B	1.00 (2)	N6A—H6A	0.91 (2)
C1B—H1B	0.9300	C1A—H1A	0.9300
C1B—C2B	1.383 (3)	C1A—C2A	1.381 (3)
C2B—H2B	0.9300	C2A—H2A	0.9300
C2B—C3B	1.347 (3)	C2A—C3A	1.351 (3)
C3B—H3B	0.958 (19)	C3A—H3A	0.9300
C4B—C5B	1.385 (2)	C4A—C5A	1.382 (2)
C5B—H5B	0.9300	C5A—H5A	0.9300
C5B—C6B	1.377 (3)	C5A—C6A	1.369 (3)
C6B—H6BA	0.9300	C6A—H6AA	0.9300
C6B—C7B	1.380 (2)	C6A—C7A	1.385 (3)
C7B—H7B	0.9300	C7A—H7A	0.9300
C7B—C8B	1.370 (2)	C7A—C8A	1.376 (2)
C8B—C9B	1.464 (2)	C8A—C9A	1.465 (2)
C10B—C11B	1.471 (2)	C10A—C11A	1.476 (2)
C11B—C12B	1.387 (3)	C11A—C12A	1.380 (3)
C11B—C16B	1.388 (3)	C11A—C16A	1.392 (2)
C12B—H12B	0.9300	C12A—H12A	0.9300
C12B—C13B	1.391 (3)	C12A—C13A	1.389 (3)
C13B—C14B	1.377 (3)	C13A—C14A	1.378 (3)
C14B—H14B	0.9300	C14A—H14A	0.9300
C14B—C15B	1.381 (3)	C14A—C15A	1.379 (3)
C15B—C16B	1.383 (3)	C15A—C16A	1.386 (3)
C16B—H16B	0.9300	C16A—H16A	0.9300
C17B—H17A	0.9600	C17A—H17D	0.9600
C17B—H17B	0.9600	C17A—H17E	0.9600
C17B—H17C	0.9600	C17A—H17F	0.9600
C18B—H18A	0.9600	C18A—H18D	0.9600
C18B—H18B	0.9600	C18A—H18E	0.9600
C18B—H18C	0.9600	C18A—H18F	0.9600
			
C13B—O1B—C17B	118.4 (2)	C13A—O1A—C17A	117.68 (17)
C15B—O2B—C18B	118.86 (19)	C15A—O2A—C18A	118.51 (17)
C1B—N1B—N2B	103.16 (18)	C1A—N1A—N2A	103.69 (17)
N1B—N2B—C4B	119.88 (16)	N1A—N2A—C4A	119.92 (16)
C3B—N2B—N1B	111.83 (16)	C3A—N2A—N1A	111.71 (17)
C3B—N2B—C4B	128.29 (16)	C3A—N2A—C4A	128.36 (17)
C4B—N3B—C8B	116.57 (15)	C4A—N3A—C8A	116.75 (16)
C9B—N4B—C10B	103.11 (14)	C9A—N4A—C10A	102.74 (15)
C10B—N5B—N6B	102.76 (14)	C10A—N5A—N6A	102.57 (15)
N5B—N6B—H6B	118.1 (12)	N5A—N6A—H6A	119.6 (12)
C9B—N6B—N5B	110.46 (14)	C9A—N6A—N5A	110.01 (16)
C9B—N6B—H6B	131.3 (11)	C9A—N6A—H6A	130.3 (13)
N1B—C1B—H1B	123.5	N1A—C1A—H1A	123.7
N1B—C1B—C2B	113.0 (2)	N1A—C1A—C2A	112.67 (19)
C2B—C1B—H1B	123.5	C2A—C1A—H1A	123.7
C1B—C2B—H2B	127.5	C1A—C2A—H2A	127.4
C3B—C2B—C1B	105.0 (2)	C3A—C2A—C1A	105.1 (2)
C3B—C2B—H2B	127.5	C3A—C2A—H2A	127.4
N2B—C3B—H3B	120.6 (12)	N2A—C3A—C2A	106.79 (19)
C2B—C3B—N2B	107.09 (19)	N2A—C3A—H3A	126.6
C2B—C3B—H3B	132.3 (12)	C2A—C3A—H3A	126.6
N3B—C4B—N2B	114.84 (16)	N3A—C4A—N2A	114.53 (16)
N3B—C4B—C5B	124.49 (17)	N3A—C4A—C5A	124.92 (18)
C5B—C4B—N2B	120.67 (15)	C5A—C4A—N2A	120.55 (17)
C4B—C5B—H5B	121.4	C4A—C5A—H5A	121.5
C6B—C5B—C4B	117.21 (17)	C6A—C5A—C4A	116.96 (18)
C6B—C5B—H5B	121.4	C6A—C5A—H5A	121.5
C5B—C6B—H6BA	120.0	C5A—C6A—H6AA	119.9
C5B—C6B—C7B	120.02 (18)	C5A—C6A—C7A	120.13 (18)
C7B—C6B—H6BA	120.0	C7A—C6A—H6AA	119.9
C6B—C7B—H7B	120.9	C6A—C7A—H7A	120.9
C8B—C7B—C6B	118.13 (17)	C8A—C7A—C6A	118.28 (19)
C8B—C7B—H7B	120.9	C8A—C7A—H7A	120.9
N3B—C8B—C7B	123.58 (16)	N3A—C8A—C7A	122.96 (18)
N3B—C8B—C9B	114.29 (15)	N3A—C8A—C9A	114.63 (16)
C7B—C8B—C9B	122.11 (15)	C7A—C8A—C9A	122.39 (18)
N4B—C9B—C8B	127.11 (15)	N4A—C9A—N6A	110.59 (16)
N6B—C9B—N4B	109.79 (14)	N4A—C9A—C8A	127.71 (17)
N6B—C9B—C8B	123.04 (15)	N6A—C9A—C8A	121.70 (17)
N4B—C10B—C11B	123.64 (16)	N4A—C10A—C11A	122.97 (16)
N5B—C10B—N4B	113.88 (15)	N5A—C10A—N4A	114.10 (16)
N5B—C10B—C11B	122.44 (16)	N5A—C10A—C11A	122.93 (16)
C12B—C11B—C10B	119.60 (17)	C12A—C11A—C10A	118.97 (17)
C12B—C11B—C16B	121.14 (18)	C12A—C11A—C16A	121.10 (18)
C16B—C11B—C10B	119.22 (18)	C16A—C11A—C10A	119.93 (17)
C11B—C12B—H12B	120.6	C11A—C12A—H12A	120.2
C11B—C12B—C13B	118.7 (2)	C11A—C12A—C13A	119.52 (18)
C13B—C12B—H12B	120.6	C13A—C12A—H12A	120.2
O1B—C13B—C12B	123.5 (2)	O1A—C13A—C12A	124.26 (19)
O1B—C13B—C14B	116.2 (2)	O1A—C13A—C14A	116.01 (18)
C14B—C13B—C12B	120.3 (2)	C14A—C13A—C12A	119.73 (19)
C13B—C14B—H14B	119.7	C13A—C14A—H14A	119.7
C13B—C14B—C15B	120.5 (2)	C13A—C14A—C15A	120.53 (19)
C15B—C14B—H14B	119.7	C15A—C14A—H14A	119.7
O2B—C15B—C14B	115.8 (2)	O2A—C15A—C14A	114.99 (18)
O2B—C15B—C16B	124.1 (2)	O2A—C15A—C16A	124.4 (2)
C14B—C15B—C16B	120.1 (2)	C14A—C15A—C16A	120.56 (19)
C11B—C16B—H16B	120.4	C11A—C16A—H16A	120.7
C15B—C16B—C11B	119.2 (2)	C15A—C16A—C11A	118.55 (19)
C15B—C16B—H16B	120.4	C15A—C16A—H16A	120.7
O1B—C17B—H17A	109.5	O1A—C17A—H17D	109.5
O1B—C17B—H17B	109.5	O1A—C17A—H17E	109.5
O1B—C17B—H17C	109.5	O1A—C17A—H17F	109.5
H17A—C17B—H17B	109.5	H17D—C17A—H17E	109.5
H17A—C17B—H17C	109.5	H17D—C17A—H17F	109.5
H17B—C17B—H17C	109.5	H17E—C17A—H17F	109.5
O2B—C18B—H18A	109.5	O2A—C18A—H18D	109.5
O2B—C18B—H18B	109.5	O2A—C18A—H18E	109.5
O2B—C18B—H18C	109.5	O2A—C18A—H18F	109.5
H18A—C18B—H18B	109.5	H18D—C18A—H18E	109.5
H18A—C18B—H18C	109.5	H18D—C18A—H18F	109.5
H18B—C18B—H18C	109.5	H18E—C18A—H18F	109.5
			
O1B—C13B—C14B—C15B	179.6 (2)	O1A—C13A—C14A—C15A	179.13 (17)
O2B—C15B—C16B—C11B	179.8 (2)	O2A—C15A—C16A—C11A	−179.40 (17)
N1B—N2B—C3B—C2B	−0.6 (3)	N1A—N2A—C3A—C2A	0.1 (3)
N1B—N2B—C4B—N3B	−163.67 (18)	N1A—N2A—C4A—N3A	−172.23 (15)
N1B—N2B—C4B—C5B	16.5 (3)	N1A—N2A—C4A—C5A	8.1 (3)
N1B—C1B—C2B—C3B	−0.6 (3)	N1A—C1A—C2A—C3A	−0.2 (3)
N2B—N1B—C1B—C2B	0.2 (3)	N2A—N1A—C1A—C2A	0.3 (3)
N2B—C4B—C5B—C6B	179.13 (19)	N2A—C4A—C5A—C6A	178.88 (17)
N3B—C4B—C5B—C6B	−0.6 (3)	N3A—C4A—C5A—C6A	−0.8 (3)
N3B—C8B—C9B—N4B	177.03 (17)	N3A—C8A—C9A—N4A	−175.45 (17)
N3B—C8B—C9B—N6B	0.1 (3)	N3A—C8A—C9A—N6A	4.8 (2)
N4B—C10B—C11B—C12B	−2.3 (3)	N4A—C10A—C11A—C12A	−13.6 (3)
N4B—C10B—C11B—C16B	−179.81 (18)	N4A—C10A—C11A—C16A	165.87 (16)
N5B—N6B—C9B—N4B	−0.3 (2)	N5A—N6A—C9A—N4A	0.0 (2)
N5B—N6B—C9B—C8B	177.14 (16)	N5A—N6A—C9A—C8A	179.81 (15)
N5B—C10B—C11B—C12B	175.01 (18)	N5A—C10A—C11A—C12A	166.35 (17)
N5B—C10B—C11B—C16B	−2.6 (3)	N5A—C10A—C11A—C16A	−14.2 (3)
N6B—N5B—C10B—N4B	0.7 (2)	N6A—N5A—C10A—N4A	−0.25 (19)
N6B—N5B—C10B—C11B	−176.76 (16)	N6A—N5A—C10A—C11A	179.83 (16)
C1B—N1B—N2B—C3B	0.3 (3)	C1A—N1A—N2A—C3A	−0.3 (2)
C1B—N1B—N2B—C4B	179.7 (2)	C1A—N1A—N2A—C4A	−178.99 (18)
C1B—C2B—C3B—N2B	0.7 (3)	C1A—C2A—C3A—N2A	0.0 (3)
C3B—N2B—C4B—N3B	15.6 (3)	C3A—N2A—C4A—N3A	9.3 (3)
C3B—N2B—C4B—C5B	−164.2 (2)	C3A—N2A—C4A—C5A	−170.40 (19)
C4B—N2B—C3B—C2B	−180.0 (2)	C4A—N2A—C3A—C2A	178.73 (19)
C4B—N3B—C8B—C7B	0.1 (3)	C4A—N3A—C8A—C7A	0.1 (3)
C4B—N3B—C8B—C9B	−178.27 (16)	C4A—N3A—C8A—C9A	−178.36 (14)
C4B—C5B—C6B—C7B	0.4 (3)	C4A—C5A—C6A—C7A	0.2 (3)
C5B—C6B—C7B—C8B	0.0 (3)	C5A—C6A—C7A—C8A	0.5 (3)
C6B—C7B—C8B—N3B	−0.3 (3)	C6A—C7A—C8A—N3A	−0.7 (3)
C6B—C7B—C8B—C9B	177.93 (18)	C6A—C7A—C8A—C9A	177.73 (17)
C7B—C8B—C9B—N4B	−1.4 (3)	C7A—C8A—C9A—N4A	6.0 (3)
C7B—C8B—C9B—N6B	−178.34 (18)	C7A—C8A—C9A—N6A	−173.67 (17)
C8B—N3B—C4B—N2B	−179.39 (16)	C8A—N3A—C4A—N2A	−179.06 (15)
C8B—N3B—C4B—C5B	0.4 (3)	C8A—N3A—C4A—C5A	0.6 (3)
C9B—N4B—C10B—N5B	−0.9 (2)	C9A—N4A—C10A—N5A	0.3 (2)
C9B—N4B—C10B—C11B	176.54 (17)	C9A—N4A—C10A—C11A	−179.80 (16)
C10B—N4B—C9B—N6B	0.7 (2)	C10A—N4A—C9A—N6A	−0.19 (19)
C10B—N4B—C9B—C8B	−176.60 (18)	C10A—N4A—C9A—C8A	−179.93 (17)
C10B—N5B—N6B—C9B	−0.27 (19)	C10A—N5A—N6A—C9A	0.12 (19)
C10B—C11B—C12B—C13B	−177.99 (19)	C10A—C11A—C12A—C13A	179.06 (16)
C10B—C11B—C16B—C15B	176.88 (19)	C10A—C11A—C16A—C15A	179.79 (16)
C11B—C12B—C13B—O1B	−179.5 (2)	C11A—C12A—C13A—O1A	−178.72 (17)
C11B—C12B—C13B—C14B	1.4 (3)	C11A—C12A—C13A—C14A	1.1 (3)
C12B—C11B—C16B—C15B	−0.6 (3)	C12A—C11A—C16A—C15A	−0.8 (3)
C12B—C13B—C14B—C15B	−1.3 (4)	C12A—C13A—C14A—C15A	−0.7 (3)
C13B—C14B—C15B—O2B	−178.9 (2)	C13A—C14A—C15A—O2A	−179.91 (17)
C13B—C14B—C15B—C16B	0.1 (4)	C13A—C14A—C15A—C16A	−0.5 (3)
C14B—C15B—C16B—C11B	0.8 (3)	C14A—C15A—C16A—C11A	1.2 (3)
C16B—C11B—C12B—C13B	−0.5 (3)	C16A—C11A—C12A—C13A	−0.4 (3)
C17B—O1B—C13B—C12B	23.7 (4)	C17A—O1A—C13A—C12A	16.8 (3)
C17B—O1B—C13B—C14B	−157.2 (2)	C17A—O1A—C13A—C14A	−163.09 (17)
C18B—O2B—C15B—C14B	−178.0 (2)	C18A—O2A—C15A—C14A	170.7 (2)
C18B—O2B—C15B—C16B	3.0 (4)	C18A—O2A—C15A—C16A	−8.7 (3)
